# Supplementary material for: Assessing the Feasibility of Providing a Family Skills Intervention, “Strong Families”, for Refugee Families Residing in Reception Centers in Serbia
Source: Int J Environ Res Public Health. 2021 Apr 24;18(9):4530. doi: 10.3390/ijerph18094530 (PMC8123170; doi:10.3390/ijerph18094530)
Supplement: Supplementary file 1 [file ijerph-18-04530-s001.zip › ijerph-1177596-supplementary.pdf]

Supplementary materials

Table S1 (within supplementary materials. Demographic Characteristics of Study Participants at Baseline as reported by caregivers.

CAREGIVERS		Total (n=25)	Mothers (n=20)	Fathers (n=5)	*t, X*^{2}	p-value
**Age of caregivers**	Mean in years (±SD)	33.4 (5.99)	32.6 (6.11)	36.8 (4.49)	t_{23}=1.45	*0.160*
**Marital status**	**Married**	22 (88%)	17 (85%)	5 (100%)	0.85 (2 df)	*0.653*
	**Divorced/separated**	1 (4%)	1 (5%)	0		
	**Widow/er**	2 (8%)	2 (10%)	0		
**Education**	**Primary School or Less**	14 (56%)	12 (60%)	2 (40%)	1.79 (3 df)	*0.618*
	**Some High School**	5 (20%)	4 (20%)	1 (20%)		
	**Completed High School**	5 (20%)	3 (15%)	2 (40%)		
	**University Degree**	1 (4%)	1 (5%)	0		
**Partner's education**	**Primary School or Less**	12 (48%)	10 (50%)	2 (40%)	4.86 (3 df)	*0.182*
	**Some High School**	9 (36%)	8 (40%)	1 (20%)		
	**Completed High School**	3 (12%)	1 (5%)	2 (40%)		
	**University Degree**	1 (4%)	1 (5%)	0		
**Working Status**	**Full Time**	1 (4%)	1 (5%)	0	3.28 (3 df)	*0.350*
	**Part Time**	6 (24%)	6 (30%)	0		
	**Searching for a job**	8 (32%)	5 (25%)	3 (60%)		
	**Not working**	10 (40%)	8 (40%)	2 (40%)		
**Partner's working status**	**Full Time**	1 (4.6%)	1 (5.9%)	0	7.03 (3 df)	*0.071*
	**Part Time**	9 (40.9%)	9 (52.9%)	0		
	**Searching for a job**	5 (22.7%)	2 (11.8%)	3 (60%)		
	**Not working**	7 (31.8%)	5 (29.4%)	2 (40%)		
**Number of Children**	Mean (±-SD)	3.3 (1.70)	3.5 (1.79)	2.6 (1.14)	t_{23}=-1.06	*0.300*
	**1**	3 (12%)	2 (10%)	1 (20%)	1.77 (4 df)	*0.778*
	**2**	5 (20%)	4 (20%)	1 (20%)		
	**3**	8 (32%)	6 (30%)	2 (40%)		
	**4**	4 (16%)	3 (15%)	1 (20%)		
	**More than 4**	5 (20%)	5 (25%)	0		
**CHILDREN in program**		**Total (n=25)**	**Girls (n=9)**	**Boys (n=16)**	***t, X*^{2}**	**p-value**
**Age of child in program**	Mean in years (±SD)	10.5 (2.38)	11.2 (2.77)	10.1 (2.11)	t_{23}=-1.18	*0.251*
**Mean age (± SD) of other children**	**Child 2** (n=22)	9.5 (3.64)	10.6 (4.39)	8.5 (2.46)	t_{20}=-1.38	*0.184*
	**Child 3** (n=17)	7.7 (3.64)	8.3 (3.20)	7.4 (3.96)	t_{15}=-0.51	*0.615*
	**Child 4** (n=9)	6.5 (5.40)	4.6 (4.82)	7.5 (5.82)	t_{7}=0.74	*0.482*
	**Child 5** (n=5)	6.8 (3.77)	6.8 (3.77)	-	-	-

	**Child 6** (n=3)	7.7 (8.08)	3.0 (-)	10.0 (9.90)	t_1=0.58	*0.667*
**Relationship to caregiver**	**Mother**	20 (80%)	9 (100%)	11 (69%)	3.52 (1 df)	*0.061*
	**Father**	5 (20%)	0	5 (31%)		
**FAMILIES**		**Total (n=25)**	**Mothers (n=20)**	**Fathers (n=5)**	***t, X*²**	**p-value**
**In Serbia**	Mean in years (±SD)	1.0 (0.14)	1.0 (0.16)	1.0 (0.07)	t_{23}=-0.23	*0.824*

* Country of origin: Afghanistan, SD: standard deviation.
